# Functional Role of Non-Muscle Myosin II in Microglia: An Updated Review

**DOI:** 10.3390/ijms22136687

**Published:** 2021-06-22

**Authors:** Chiara Porro, Antonio Pennella, Maria Antonietta Panaro, Teresa Trotta

**Affiliations:** 1 Department of Clinical and Experimental Medicine, University of Foggia, 71121 Foggia, Italy; chiara.porro@unifg.it (C.P.); antonio.pennella@unifg.it (A.P.); 2 Department of Biosciences, Biotechnologies and Biopharmaceutics, University of Bari, 70125 Bari, Italy; mariaantonietta.panaro@uniba.it

**Keywords:** non-muscle myosin II, microglia, cytoskeleton, phagocytosis, migration, cell morphology

## Abstract

Myosins are a remarkable superfamily of actin-based motor proteins that use the energy derived from ATP hydrolysis to translocate actin filaments and to produce force. Myosins are abundant in different types of tissues and involved in a large variety of cellular functions. Several classes of the myosin superfamily are expressed in the nervous system; among them, non-muscle myosin II (NM II) is expressed in both neurons and non-neuronal brain cells, such as astrocytes, oligodendrocytes, endothelial cells, and microglia. In the nervous system, NM II modulates a variety of functions, such as vesicle transport, phagocytosis, cell migration, cell adhesion and morphology, secretion, transcription, and cytokinesis, as well as playing key roles during brain development, inflammation, repair, and myelination functions. In this review, we will provide a brief overview of recent emerging roles of NM II in resting and activated microglia cells, the principal regulators of immune processes in the central nervous system (CNS) in both physiological and pathological conditions. When stimulated, microglial cells react and produce a number of mediators, such as pro-inflammatory cytokines, free radicals, and nitric oxide, that enhance inflammation and contribute to neurodegenerative diseases. Inhibition of NM II could be a new therapeutic target to treat or to prevent CNS diseases.

## 1. Introduction

Myosins are a superfamily of actin-based motor proteins that use the energy derived from ATP hydrolysis to translocate actin filaments and to produce force. In humans, the myosin superfamily consists of 40 myosin genes, which are classified into 12 different classes on the basis of their architecture; a single cell can express multiple classes and splice forms of myosins [1,2].

Myosin II, or conventional myosin, was the first myosin discovered; it has been isolated from the thick filaments of muscle and is responsible for skeletal muscle contraction, while the isoforms of this “conventional” myosin would be recognized as also responsible for cardiac and smooth muscle contraction [3]. All subsequently identified myosins have been compared to Myosin II.

In 1973, Pollard and Korn identified and isolated the first unconventional myosin subfamily from the soil amoeba *Acanthamoeba castellanii*; it was called Myosin I because it was the first myosin to be isolated from Acanthamoeba [4]. It was also the first single-headed myosin, consisting of a single relatively small heavy chain (~125 kDa), and did not form filaments, in contrast to conventional Myosin II. Myosin I is the largest unconventional myosin subfamily, and higher vertebrates express eight different Myosin I genes (Myo1a–Myo1h) [5]. Following that, other unconventional members of the myosin superfamily were identified [5,6].

Myosins can generally be grouped into two major categories: conventional myosins, which include the skeletal, cardiac, and smooth muscle myosins, and the non-muscle myosin II (NM II), with their C-terminal tails containing a characteristic long coiled-coil for bipolar filament formation, and unconventional myosins, with diverse tails [6,7].

In addition to being present in striated and smooth muscle cells, myosins are also abundant in other different cell types, such as granulocytes, platelets, fibroblasts, neurons, and glial cells, where they are involved in a wide variety of cellular functions [5,6,7,8,9]. The different myosin classes modulate actin-based fundamental cellular processes, including vesicle transport, cell migration, cell-substrate interactions, phagocytosis, secretion, endocytosis, exocytosis, intracellular tracking, organelle and plasma membrane morphology, cell adhesion, and maintenance of cortical tension. Moreover, classes of myosins found in the nucleus participate in nuclear functions, including transcription, chromatin remodeling, and chromosome movement [7,8,10,11,12].

Several classes of the myosin superfamily (class I, II, III, V, VI, VII, IX, X, XV, and XVI) are expressed in the nervous system, where they play essential roles in diverse biological functions involved in the development and functioning of neural cells, such as neuroblast differentiation, neuronal migration, growth cone motility, axonal growth, and transport or synaptic functions [13,14].

In the central nervous system (CNS), evidence shows that NM II regulates actin filament cytoskeleton assembly and contractile forces essential for not only neuronal development and function but for many non-neuronal cell functions. In fact, in glial cells such as microglia, astrocytes, and oligodendrocytes, NM II isoforms perform key functions in biological processes, including inflammation, myelination, repair and blood–brain barrier (BBB) integrity [15,16,17,18]. In this review, we will focus on the emerging roles of NM II in resting and activated microglia cells, the resident immune cells of CNS.

## 2. Non-Muscle Myosin II

In humans, class II myosins are heterogenous in structure and function [19]. They include sarcomeric skeletal (Myh1, Myh2, Myh3, Myh4, Myh8, Myh13, Myh15), cardiac (Myh6, Myh7, Myh7b), and non-sarcomeric smooth muscle (Myh11, with two splice variants) myosins, as well as NM II isoforms that, in their respective cells, self-assemble into bipolar/side-polar filaments, forming dynamic flexible contractile structures [1,20].

**Figure 1:**
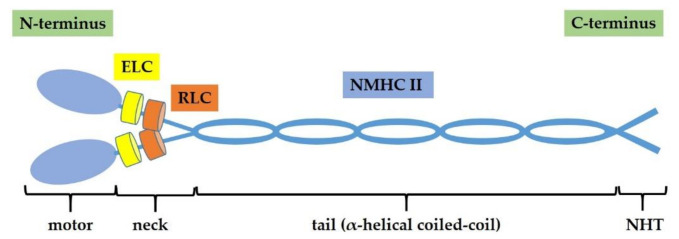
Non-muscle myosin II (NM II) structure. NM II is a hexamer composed by two non-muscle heavy chains (NMHC IIs) of 230 kDa, two regulatory light chains (RLCs) of 20 kDa, and two essential light chains (ELCs) of 17 kDa. NM II complex consists of three regions: motor domain, neck domain, and tail domain (α-helical coiled-coil that terminates in a non-helical tailpiece, NHT).

NM II protein is a hexameric complex composed by two non-muscle heavy chains (NMHC IIs) of 230 kDa, two regulatory light chains (RLCs) of 20 kDa and two essential light chains (ELCs) of 17 kDa. Myosin light chains (MLCs) includes RLCs and ELCs [21]. The NM II complex can be structurally divided into three regions: the motor or head, the neck, and the tail domains. The *N*-terminus of each heavy chain contains the conserved catalytic head domain, where the ATP and actin binding sites are located, thus, it is responsible for actin-filament binding and ATP hydrolysis. NMHC IIs are connected by long α-helical coiled-coil regions at the C-terminus that constitutes the tail, particularly important for the proper subcellular localization of the different NM II isoforms. The coiled-coil domain terminates in a non-helical tailpiece (NHT). The light chains associate with the heavy chains in the neck domain, which serve as a mechanical lever to amplify force generated in the head domain [14,22] (Figure 1).

In mammalian cells, three different heavy chain genes present on different chromosomes and encoding three NM II isoforms were discovered: Myh9 (NM IIA), Myh10 (NM IIB), and Myh14 (NM IIC) [14]. The three myosin II isoforms were found in non-muscle mammalian cells, where they have been implicated in several processes, including cell migration, cytokinesis, cell adhesion, and tissue morphogenesis [1,23,24], and participate in human diseases ranging from cancer to neurological disorders [21,25].

The motor domain is highly conserved between the different NM II isoforms, particularly in the actin-binding site; in contrast, both the C-terminal rod and the non-helical tail differ significantly among the three isoforms. In fact, despite their high conservation at the sequence and structural level, NM II isoforms display significant differences regarding the dynamics of the myosin filament assembly, ATPase activities, duty ratios (the fraction of time the myosin head spends strongly bound to actin during an ATPase cycle) and intracellular location, determining specific functions in the cells [19,26,27,28].

In their active conformation, NM II molecules interact antiparallelly by their tail regions and self-associate into NM II bipolar filaments with the motor domains oriented to the outside of the polymer [20,29,30].

NM II motor activity, activation, and assembly state are determined by the phosphorylation of both the NMHC and the associated RLC. NMHC II phosphorylation is essential for filament formation and NM II regulation in amoeba [31], but the consequences of NMHC II phosphorylation are still being defined in mammals. RLC phosphorylation stimulates the actin-activated ATPase activity and promotes myosin filaments assembly. Phosphorylation of RLC on Serine 19 and Threonine 18 is a reversible biochemical process tightly regulated by several myosin kinases and phosphatases, such as MLC-kinase (MLCK), Rho-associated protein kinase (ROCK), leucine-zipper-interacting protein kinase (ZIPK), citron kinase, serine/threonine-protein kinase (PAK), and myotonic dystrophy kinase-related CDC42-binding kinase (MRCK/CDC42BP) [32,33,34,35,36,37,38] (Table 1). These kinases display specific intracellular locations and are modulated by a variety of signal transduction pathways that provide an intricate regulation to accurately modulate NM II activity. NM II isoforms are primarily regulated by phosphorylation of RLC Serine 19 via calcium–calmodulin-dependent MLCK or ROCK [39], a serine/threonine kinase that phosphorylates MLC directly or/and via the inhibition of myosin light chain phosphatase (MLCP) [40].

Each NM II isoform displays different tissue/organ expression and intracellular distribution; generally, NM IIA and NM IIB are the predominant isoforms [14]. Nevertheless, they also have redundant and interchangeable functions, as demonstrated by the fact that all NM II isoforms are able to proficiently support cytokinesis [41].

Even if NM IIB is the predominant isoform in neurons, the expression of all three isoforms, NM II, has also been described [42,43] as essential for neuronal biology, growth of axons during development and axon regeneration in the adult, radial and longitudinal axonal tension, dendritic spine, synapse morphology, and plasticity [14,44,45,46].

In addition to its classical functions in neurons, NM II plays important roles in glial cell biology processes. In astrocytes, all three isoforms are similarly expressed [47]. NM II is important for BBB integrity and permeability [15] and is involved in astrocyte polarization and migration towards the injured tissue in response to brain damage [48]. Conversely, in oligodendrocytes, NM IIC was reported to be the most abundant isoform [47], instead NM IIA and NM IIB were identified as negative regulators of oligodendrocyte maturation and myelination [49,50] and are expressed also in endothelial cells, where they regulate brain vasculature [51]. Lastly, NM IIA was reported to be the most abundant isoform in microglia [47], but it is important to note that Stefanie Janßen et al. demonstrated NM IIB expression in activated microglia during experimental demyelination of the CNS [18].

## 3. Non-Muscle Myosin II in Microglia

### 3.1. Microglia

Microglia are the resident phagocytic cells of the CNS and are the principal regulators of immune processes in the brain both in physiological and pathological conditions [55,56]. Microglia are closely related to CNS macrophages but still represent a distinct specialized population of tissue macrophages [57]; the precursor cells of microglia reach the CNS during embryogenesis and proliferate further during the neonatal period. Further recruitment of microglia precursor cells from the circulation in adulthood occurs only under unique conditions [55,58].

The functional spectrum of microglia responses is broad; they exert immunoregulatory and repair functions and are required for removal of dead cell debris and abnormally accumulated proteins [59]. In the developing brain, microglia display “amoeboid” morphology and phagocytic activity for the removal of apoptotic cells and the modeling of CNS neuronal circuits [60]. Furthermore, these cells are essential for survival and proliferation of neurons and for the maintenance of neuronal plasticity [55,61].

In the adult CNS, under normal homeostasis, microglia are associated with an immunosurveillance state and maintenance of homeostasis. Resting microglia are characterized by a ramified morphology with long cytoplasmic protrusions that continuously scan their microenvironment to sense the presence of abnormal signals without moving, and their phagocytic activity subsides. Moreover, through their processes, microglia make specific and repeated cell–cell contacts with various cell types and, more intimately, with neurons [62,63].

However, as a result of injury and disease, microglia activate, proliferate, and change their morphology with an increase in cell body size and short and robust cell processes. In some cases, microglia revert to a complete amoeboid phagocytic morphology [64] and move and accumulate into the damaged area, and their phagocytic activity resumes, enabling them to remove tissue debris, apoptotic cells, and misfolded proteins [65,66]. Microglia can promote neuroinflammation and neurotoxicity, and for this reason, these cells have been implicated in almost all CNS disorders and in the progression of neurodegenerative diseases such as Alzheimer’s disease (AD), Parkinson’s disease (PD), and multiple sclerosis (MS) [61,67,68,69].

The activated microglia can be classified into at least two types according to their function, similarly to macrophages: as pro-inflammatory and neurotoxic (M1) or anti-inflammatory and neuroprotective (M2) type microglia. However, it is important to note that classification of microglia into either an M1 or M2 polarized state may turn out to be an oversimplification, because microglia show high levels of diversity and plasticity [70].

M1 microglia are characterized by an amoeboid shape, which is consistent with a state of hyperactivity, high mobility, and strong phagocytic capacity. These cells are associated with the production and release of several pro-inflammatory mediators, such as nitric oxide (NO); interleukin (IL)-6, IL-23, IL-1β, IL-12, and tumor necrosis factor-alpha (TNF-a); and reactive oxygen species (ROS), in order preserve brain structural and functional integrity [66,70,71].

In contrast, M2 microglia are characterized by a typical elongated morphology with branched processes and secrete anti-inflammatory molecules, such as transforming growth factor (TGF), IL-4, IL-10, IL-13, vascular endothelial growth factor (VEGF), and brain-derived neurotrophic factor (BDNF) [66,71,72,73]. Whether microglia play a beneficial or harmful role depends on many factors, such as the type of stimulus and the duration of an impact; as probably occurs in neurodegenerative diseases, a transient activation of microglia is generally neuroprotective, but chronic or prolonged reactivity of these cells can induce the M1 activation, contributing to neuronal damage [66].

Finally, emerging evidence indicates that the activation of microglia may be associated also to release of extracellular vesicles (EVs) from the cell plasma membrane into the pericellular space where they can function as cargo for delivering cellular components to other cells [74,75]. The classification of the vesicle subtypes is ongoing and includes apoptotic bodies (800–5000 nm in diameter), microparticles/microvesicles (MPs/MVs) (100–1000 nm), and exosomes (40–120 nm) [76]. In CNS diseases, some evidence shows that EVs are able to play a dual role: on one hand, cells use EVs to remove toxic proteins and aggregates from their cytoplasm; on the other, these EVs can interact with healthy cells, delivering their toxic cargoes and spreading diseases [69,77].

To perform their functions, microglia undergo a series of vigorous cellular rearrangements that require the contribution of the cytoskeleton, and NM II has been shown to play an important role in membrane and cytoskeletal remodeling during the biological processes of microglia [18].

### 3.2. NM II in Microglia Morphology and Polarization

Upon activation, microglia can undergo profound morphological changes, from hyper-ramified to amoeboid shape, which have been associated with different functional states of cells. The contractile system in non-muscle cells is highly dynamic and plays important roles not only in cell motility but also in cell shape determination [24,78]. In resting microglia, NM IIB protein is distributed diffusely, with the major expression at the center of the cell, and forms strand-like structures that extend towards the distal edges of the cell. Stefanie Janßen et al. found that, in lipopolysaccharide (LPS)-activated microglia, these structures are lost, and NM IIB is redistributed predominantly to the perinuclear area. Moreover, pharmacological treatment of microglia with the NM II inhibitor, blebbistatin, demonstrated the involvement of NM II activity in determining cell shape; indeed microglia treated with blebbistatin did not adopt the characteristic elongated structures and exhibited irregular cytoskeletal structure [18].

Various actin-myosin-related processes, such as cell motility, adhesion, phagocytosis, and morphological changes, are promoted by ROCK activation [79,80,81,82]. There is strong evidence that inhibition of ROCK activity changes the microglial phenotype, which contributes to the neuroprotective effects observed after ROCK inhibition. In a rat model of neuropathic pain, ROCK inhibition prevented morphological changes, such as retraction of processes in microglia and microglia–neuron interactions, contributing to the prevention of neuropathic pain [83]. In another research article, RhoA/ROCK-inhibited microglia exhibited an altered branched morphology with relatively thick branches distinct from fine filopodia and disruption of stress fibers [84].

Reversion of over-activated to the neuroprotective microglia phenotype could regenerate a healthy CNS-supporting microglia environment. Changes in cell morphology have been associated with different functional states of cells, and cell shape appears to play an important role in modulation of the phenotypic polarization of macrophages, orienting towards an M1 or M2 phenotype. As demonstrated by McWhorter et al., in macrophages, the elongation itself, without exogenous cytokines, leads to the expression of M2 phenotype markers and reduces the secretion of inflammatory cytokines. Furthermore, shape- but not cytokine-induced polarization is abrogated when actin and actin/myosin contractility were inhibited by pharmacological agents, suggesting a role for the cytoskeleton in the control of macrophage polarization by cell shape [85].

Although it is widely accepted that cytokine signaling elicits a polarized microglia response [86,87], probably, as seen in macrophages, cell shape itself is able to orientate microglia towards a M1 or M2 phenotype. It is interesting to note that advanced glycation end products (AGEs) could increase the level of MLC phosphorylation. Jingkao Chen et al. investigated the mechanism of AGEs/receptor of AGEs (RAGE)/Rho/ROCK pathway underlying the non-specific inflammation and microglial polarization in BV2 cells. AGEs could activate ROCK pathway in a concentration-dependent manner and induced both M1 and M2 phenotype in BV2 cells. Following treatment with AGEs, BV2 cells became activated with a greatly enlarged cell body and the characteristic shapes of activated microglia. In addition, pretreatment with fasudil or RAGE inhibitor FPS-ZM1 inhibited AGE-induced phosphorylation of MLC, blocking activation of BV2 cells and significantly promoting the polarization of M1 phenotype to M2 phenotype [88].

Recently, several studies showed that MVs released from pro-inflammatory microglia contribute to neuroinflammation and play an important role in neuronal functionality in the context of neurological disorders [67,75,89].

Cancer cell studies have provided information on the mechanism of MV biogenesis in nucleated cells [90,91]. Upon stimulation, the influx of Ca^2+^ results in the activation of Ca^2+^-dependent proteases. This, in turn, disrupts the membrane cytoskeleton with formation of membrane protrusions and phosphatidylserine exposition to the external leaflet [92,93]. The main mediators of MV biogenesis seem to be the GTPases of the Ras superfamily; in particular, activated RhoA induces the actin–myosin contraction required for MV formation [94].

In CNS, the main MVs studied are released by microglia and astrocytes upon ATP activation of P2X7 receptor [74] that is highly expressed on inflammatory cells [95]. In microglial cells, the ATP receptor P2X7 is located in raft domains [96]. Upon stimulation, it leads to enzyme acid sphigomyelinase activation with rapid sphingomyelin hydrolysis and MV formation [97]. Gu et al. showed a close molecular interaction between the P2X7 receptor and NMHC IIA. In innate and transfected monocytic cells, the human P2X7 membrane complex contained cytoskeletal proteins, including actin and NMHC IIA [98].

### 3.3. NM II in Microglia Migration

During the process of polarization toward a pro-inflammatory phenotype, microglia undergo progressive modifications, including acquisition of an amoeboid morphology, that involve a rearrangement of the cytoskeleton. Activated microglia exhibit a directional migration to the lesion site using a chemotactic gradient and, upon arriving at the injury site, undergo further transformation, becoming phagocytes [99].

Although, in vitro, several studies have proposed some mechanistic and signaling events underlying microglia chemotaxis/migration [100], in vivo, they are still largely unknown.

Initially, the migration process is characterized by the formation of membrane protrusions (lamellipods, filopods) at the cell lamella and the formation of cell-matrix adhesions near the tips of the protrusions and, subsequently, by the release of cell-substrate adhesions at the rear end of cell and contraction of cell body [101]. Several cell matrix adhesions have been identified [102]. Primary rat microglia form podosomes: tiny, highly dynamic multi-molecular adhesion structures with an F-actin-rich nucleus surrounded by an adhesion ring and structural proteins [103], which can both adhere to and degrade the extracellular matrix, allowing invasion of tissues [104].

At the front of polarized microglia, the directional incorporation of the actin monomers into the actin network generates a pushing force against the plasma membrane, which in turn, provides a resistive force. As a result, the force of actin assembly causes the entire actin network to be pushed back from the membrane in a process called retrograde actin flow. To guide the forward protrusion of the plasma membrane, the actin network must anchor itself to the substrate to prevent it from sliding back. NM II regulates protrusion and cell migration; it is present in actin stress fibers in the rear of the cell and may play an important role in coordination of cell migration and directional motility by preventing formation of lateral pseudopods and influencing the net rate of cellular protrusion. Indeed, NM II appears to generate a retrograde flow, which counteracts the actin polymerization-mediated advancement of the leading edge, reducing the protrusion rate [24,105].

The motility, as well as shape changes of microglial cells, is regulated by intracellular signals through various signaling cascades, including receptors and kinases. Xiaoxu Zhang et al. showed that RhoA/ROCK signaling, the fundamental mediator of cell movement, regulated Aβ-induced microglial chemotactic migration, as confirmed by treatment with fasudil and Y27632 that significantly suppressed Aβ-induced cell migration [106].

In a recent study by Pei-Cai Fu et al., microglia treated with ROCK inhibitors showed a larger size, an irregular shape and small processes, and changes in morphology associated with increased migration activity. In addition, extracellular-signal-regulated kinase (ERK) signaling was involved in the migratory and morphological changes observed after ROCK inhibition [82].

Cdc42 acts as a center of cell polarity and is intimately associated with the contractility and rearrangement of the actomyosin cytoskeleton and the phosphorylation of myosin phosphatase target subunit-1 (MYPT-1) at threonine 696. In vivo, inhibition of ROCK/Cdc42-mediated microglial motility blocks the activating features of microglia, such as increased cell size and number of filopodia, and diminishes their phagocyting/secreting domains, confirming Cdc42 involvement in microglial motility [107].

Honghong Yao et al. demonstrated the involvement of non-muscle MLCK (NMLCK) in microglia migration mediated by Tat, the HIV transactivator of transcription, released from infected cells in the hippocampus. In particular, the authors assessed that Tat-mediated engagement of the vascular endothelial growth factor receptor type 1 stimulated activation of NMLCK, followed by inside-out activation of β1 integrin, and subsequent outside-in signaling of β1 integrin involving activation of the downstream kinases c-Src and Pyk2 and activation of Cdc42-GTP. This mechanism resulted in actin polymerization, thereby reinforcing the integrin–cytoskeleton connection and leading to microglial migration [108].

### 3.4. NM II in Microglial Phagocytosis

Microglia provide a line of defense similarly to the function of macrophages in peripheral tissues. The fast engulfment and clearance of cell debris, myelin debris or dead cells are necessary for CNS homeostasis, avoiding inflammation and/or autoimmune response [109,110,111]. However, although initially, microglial phagocytic capacity was supposed to be exclusively related to pathological conditions, emerging findings demonstrated that it is critically involved in physiological processes [112,113], such as axon pruning and stabilization of dendritic spines for the restructuring of neuronal connections [114,115].

The regulation of microglial cell phagocytosis is orchestrated by specialized and tightly controlled mechanisms that comprise find-me, eat-me, and digest-me steps [112]. The myosin/actin network plays a critical role in the engulfment step of phagocytosis; during phagocytic clearance, NM II is redistributed and co-localizes with cargo derived from ingested cellular debris [113].

Formation of protrusions around large particles implies that substantial morphological rearrangements and mechanical forces generated by the cytoskeleton drive these structural changes. Multiple models suggest that a protrusive force is required to deform the phagocytic cell around the particulate target and to initiate formation of the phagocytic cup [115,116]. Compelling evidence indicate that, during phagosome formation and advancement, the branched actin network of the lamellipodium generates high protrusive forces to overcome the increasing surface tension [117], increasing the F-actin density and resistance under higher loads [118,119]. Protrusion of the phagocytic cup requires existing F-actin to disassemble and new F-actin to assemble in a new configuration, causing plasma membranes to protrude [120,121]. NM II does not appear to play a role in the advancement of the phagocytic cup but may participate in other aspects of phagocytosis. Indeed, it would seem that NM II-mediated contractile activity is required during phagocytic cup assembly, squeezing, and closure [122,123]; moreover, it also promotes actin disassembly at the rear of migrating cells [124]. Since myosin II activity increases cortical tension [125], it would impede protrusion around the particle but would facilitate inward movement of the particle [115]. The polarity of the actin at the leading edge and the directionality of the myosin motors result in myosin pulling actin in the opposite direction of the leading edge protrusion to drive actin retrograde flow [126,127]. The effect of myosin II inhibition might, thus, be variable for different phagocytes since they exhibit distinct cortical tensions [128].

Recently, the Rho family of small GTPases have been shown to regulate Fc receptor-mediated phagocytosis by controlling the different steps of membrane and actin dynamics, leading to particle engulfment. ROCK is activated directly by small GTPase RhoA, and RhoA/ROCK signaling is required for stress fiber assembly and maintenance, as confirmed by Miri Gitik et al. The authors investigated the phagocytosis of degenerated myelin and zymosan by primary microglia and demonstrated that it depends on the cytoskeletal MLCK and Rho/ROCK signaling, which can activate or inhibit phagocytosis and, therefore. change roles based on the nature of the phagocytosed particle and the receptors that mediate each phagocytosis [84]. The use of inhibitors suggested that Rho/ROCK, but not MLCK, regulates F-actin assembly, disassembly, and stabilization. In addition, ROCK can further upregulate actomyosin contractility by downregulating MLCP [84].

In an experimental mouse model of PD following the intraperitoneal injection of 1-Methyl-4-phenyl-1,2,3,6-tetrahydropyridine (MPTP), ROCK/Cdc42 signaling mediates phagocytosis of degenerated dopaminergic neurons, whereas ROCK inhibition prevents it [107], suggesting that inhibition of RhoA/ROCK-mediated actomyosin activation may be beneficial for reducing neuron loss in neurodegenerative diseases [40]. Indeed, previous studies have shown that microglia phagocytose viable neurons in a neuro-inflammatory environment and induce apoptosis; moreover, dopaminergic neurons are especially vulnerable to inflammation [129,130]. In a recent study, Scheiblich and Bicker demonstrated that inhibition of RhoA/ROCK signaling significantly reduced the engulfment of neuronal debris by reactive microglia, while the engulfment by resting microglia was not affected. As a result, the authors argue that inhibition of RhoA/ROCK may represent a therapeutic strategy for the treatment of excessive inflammation and neurodegeneration induced by microglia in the CNS [79].

Ras-related C3 botulinum toxin substrate 1 (Rac1), a protein of the Rho family GTPases, regulates both neuronal apoptosis and actin cytoskeleton [131]. Rac1-MLCK signaling pathway is involved in the increased P2Y6R-mediated microglial phagocytosis observed in radiation-induced brain injury [132]. The Rac1 inhibitor, NSC23766, suppressed expression of MLCK, indicating that the Rac1-MLCK pathway was involved in microglial phagocytosis [133].

Phagocytosis involves membrane and cytoskeletal rearrangements, which is a highly energetic process requiring hydrolysis of ATP by non-muscle myosin. In the absence of ATP, the P2X7 complex is anchored to actin cytoskeleton via NMHC to mediate phagocytosis. Following the binding of ATP, the physiological agonist of P2X7, NM dissociates from the P2X7 complex, leading to loss of phagocytic ability. Interestingly, actin polymerization and myosin activity blockers inhibit P2X7-mediated phagocytosis [134]. Probably, also in microglia, NM II/actin cytoskeletal dynamics participate in P2X7-mediated phagocytosis, as suggested by the study on innate P2X7-mediated phagocytosis of blood monocytes that was decreased in AD patients showing more amyloid pathology [135,136]. A more recent study assessed that the microglia expression of P2X7 confers a phagocytic ability on the cell, and an attachment to NM of the cytoskeleton is required for particle engulfment [137].

## 4. Conclusions

NM II is a central protein in cell mechanics and plays a key role in a variety of important cell biology processes. As discussed in this review, in microglial cells, NM II is involved in the remodeling of the membrane and cytoskeleton necessary for the performance of functions that characterize activated microglia, such as polarization, migration, and phagocytosis.

Microglia are essential in maintaining homeostasis and normal CNS function, both during development and in response to injury. Paradoxically, these cells, although crucial for the host defense, can contribute to neuropathology; much research has confirmed that the over-activation of microglia can enhance neuroinflammation, contributing to neuronal damage and the onset and progression of some neurodegenerative diseases. Therefore, modulation of microglia activation towards the neuroprotective phenotype has been proposed as a novel therapeutic strategy for patients with neurological disorders.

An interesting therapeutic method could be to act on NM II, preventing an excessive activation of microglia. Recently, a large number of pharmacological NM II inhibitors have been developed, which can affect NM II functions in two ways. The first is to modulate NM II expression at the post-transcriptional level using new NM II inhibitors (such as microRNA and siRNA); however, potential toxicity and off-target effects limit their application. The second is to suppress NM II activity via inhibition of ATPase, MLCK, or other kinases such as ROCK. However, one of the major drawbacks of kinase pharmacological inhibition is the incomplete specificity of the inhibitors towards the target, which often implies significant regulation of other kinases, leading to off-target effects.

It has become increasingly evident that the various regulatory mechanisms do not act in isolation, but there is a significant interaction between them. Our current knowledge reveals the existence of a very complex regulatory network that contributes to the fine regulation of NM II activity in different cellular contexts and in response to a variety of stimuli. Consequently, further research is needed to identify and understand the molecular mechanisms that control NM II in microglia. Future studies could provide critical insights that may lead to the development of new therapeutic strategies for the treatment of CNS diseases.

## Figures and Tables

**Table 1:** Regulation of NM II. Several kinases regulate the activation/inactivation state of NM II through the phosphorylation of different Ser and Thr residues. Myosin light chain kinase (MLCK), Rho-associated protein kinase (ROCK), citron Rho-interacting kinase (CRIK), death-associated protein kinase (DAPK3), myotonic dystrophy-related Cdc42-binding protein kinase (MRCK), leucine-zipper-interacting kinase (ZIPK), p-21 activated kinase (PAK), casein kinase II (CKII), protein kinase C (PKC).

Effect on NM II.	Kinases	References
Activation at Thr18 and Ser19	MLCK	[39]
	ROCK	[39]
	CRIK	[32]
	DAPK3	[33]
	MRCK	[38]
	ZIPK	[33]
Activation at Ser19	PAK	[35]
Activation at Ser1943	CKII	[21,52]
Activation at Ser1916 and Ser1937	PKC	[21,52,53]
Inactivation at Thr9, Ser1 and Ser2	PKC	[21,54]

## Data Availability

Not applicable.
